# Circadian Rhythms in Dinoflagellates: What Is the Purpose of Synthesis and Destruction of Proteins?

**DOI:** 10.3390/microorganisms1010026

**Published:** 2013-09-18

**Authors:** J. Woodland Hastings

**Affiliations:** Department of Molecular and Cellular Biology, Harvard University, Cambridge, MA 02138, USA; E-Mail: Hastings@FAS.Harvard.edu; Tel.: +1-617-495-3714; Fax: +1-617-495-9956

**Keywords:** dinoflagellates, bioluminescence, circadian rhythms

## Abstract

There is a prominent circadian rhythm of bioluminescence in many species of light-emitting dinoflagellates. In *Lingulodinium polyedrum* a daily synthesis and destruction of proteins is used to regulate activity. Experiments indicate that the amino acids from the degradation are conserved and incorporated into the resynthesized protein in the subsequent cycle. A different species, *Pyrocystis lunula*, also exhibits a rhythm of bioluminescence, but the luciferase is not destroyed and resynthesized each cycle. This paper posits that synthesis and destruction constitutes a cellular mechanism to conserve nitrogen in an environment where the resource is limiting.

## 1. Introduction

In many organisms, ranging from bacteria to mammals, biological processes are regulated on a daily basis, such that maximum activity occurs at one time of the day. Such rhythms continue to occur after the organism is placed in constant conditions (e.g., light and temperature), but the length of the cycle is typically not exactly 24 h. This indicates the rhythmic mechanism is endogenous, and is referred to as the circadian clock (*circa*, about; dian, one day). In a 24 h cycle of light and dark, rhythms are said to be entrained, so exhibit exact 24 h periods.

Photosynthetic dinoflagellates figured prominently in early studies of circadian rhythms; the photosynthetic capacity peaks during subjective day while bioluminescence is greatest during night phases. Early studies of the circadian clock in dinoflagellates were greatly facilitated by the light emission, as it provides a built-in reporter.

It seems biologically reasonable to regulate bioluminescence, since it would be of no value during the day in the many species living near the surface. However, many other processes and proteins are similarly regulated, some with no evident functional importance. 

Bioluminescence is a feature of many but not all dinoflagellates, including both photosynthetic and heterotrophic species. A comprehensive and authoritative review of the many aspects of dinoflagellate bioluminescence is provided in this issue [1]. Many different organisms are luminous, from bacteria to fish, but with different genes and proteins; bioluminescence is thus a result of convergent evolution [2,3,4].

## 2. Many Quasi-Independent Oscillators in Single Cells

Circadian control in dinoflagellates is different from that in most other organisms, in that the rhythms are regulated at the translational level [5]. Bioluminescence itself exhibits two different rhythms, spontaneous flashing and a constant glow, the two having different times at which their peaks (acrophases) occur. Other rhythms, such as photosynthetic capacity and the time of day at which cells divide, have still other acrophases. Rhythms in the synthesis rates of several different enzymes have also been reported [6].

It has been postulated that each different rhythm has its own quasi-independent oscillator [7]; this is based upon the observation that the acrophase relationships of these can differ under different entraining conditions, such as the photo-fraction in a light-dark (LD) 24-h cycle (e.g., LD6:18 or LD18:6), or the length of the entraining period (referred to as T; e.g., 23, 24 or 25 h). Even more persuasive is the fact that in constant conditions the different rhythms may have different free-running periods [8], which cannot be readily accommodated in a single oscillator model. The different rhythms appear to be coupled, albeit more or less loosely, but the coupling mechanism is not known.

## 3. Protein Synthesis and Destruction

Reflecting the *in vivo* rhythm in *L. polyedrum*, both the luciferase (LCF) and the luciferin binding protein (LBP) are destroyed at the end of each subjective night and synthesized anew before subsequent activity peaks [9,10]. Why is such a mechanism used to regulate activity? On the face of it, one might expect a more conservative method, such as phosphorylation and dephosphorylation. 

**Figure 1:**
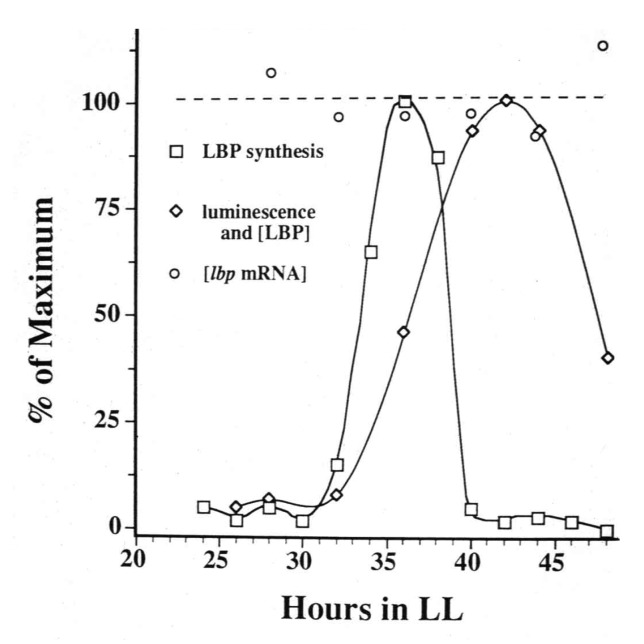
Measurements over time of the synthesis rate of luciferin binding protein (LBP) (squares, first peak), its abundance (diamonds) and the abundance of *lbp* mRNA (circles, dotted line), with cultures maintained in constant conditions.

Regulation of expression is usually associated with new transcription, but this was not observed; for LBP an approximately 5-h bout of synthesis occurred each cycle while the mRNA abundance for that protein remained constant (Figure 1; [5,11]).

**Figure 2:**
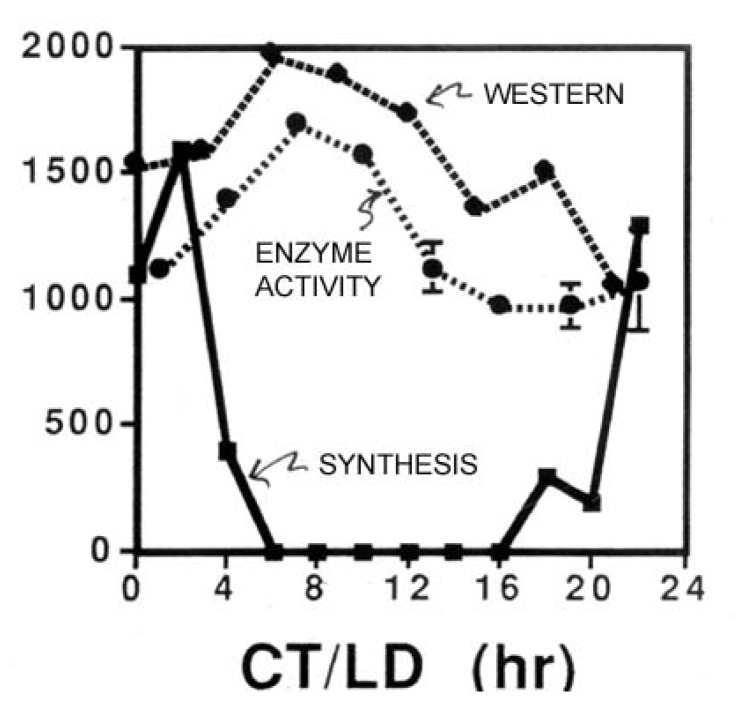
Measurements (ordinate) over time in constant conditions of the enzyme activity, abundance (Western blots) and synthesis rate of the enzyme glyceraldehyde dehydrogenase (GAPDH).

While also rhythmic, the enzyme glyceraldehyde dehydrogenase (GAPDH) illustrates an additional point of interest in circadian biology [12]. The amplitude of its abundance rhythm is not as great as it is with LBP or LCF (Figure 2), meaning that not all of the GAPDH is destroyed in a single cycle. Thus, synthesis might be strongly circadian (*i.e.*, synthesis occurs only during a short time-window), but if the protein has a long lifetime (many days), the synthesis burst would be only a small fraction of the total cellular amount of that protein, so its abundance would not exhibit a high amplitude rhythm.

Many other proteins in *L. polyedrum* exhibit circadian changes in abundance, circadian-controlled at the translational level [13]. However, equal amounts of those proteins were found to be synthesized by *in vitro* translation from poly(A)+ RNA extracted from cells at day and night phases, indicating that the message itself is not only present, but fully capable transcriptionally at all times of the cycle.

Markovic *et al.* [6] tracked the *in vivo* synthesis rates of several of these circadian-controlled proteins, later identifying several of them, and found that they fell into three acrophases, the first occurring during the late day/early night phase, the second during the middle of the night phase, and the third during the late night/early day phase (Table 1). All have high synthesis-rate amplitudes.

**Table 1:** Identities and acrophases of proteins exhibiting circadian controlled rates of synthesis.

Protein MW	Acrophase	ID
75 kDa	Early night	LBP
135 kDa	Early night	Luciferase
21 kD	Late night	Unidentified dinoflagellate protein
55 kDa	Late night	Rubisco
32 kD	Early day	PCP
33 kDa	Early day	Oxygen evolving enzyme 1
45 kDa	Early day	GAPDH

## 4. Why Protein Synthesis and Destruction?

What might be the selective advantage for daily synthesis and destruction of protein, which is surely expensive energetically? The proteins involved are not only those responsible for bioluminescence, so there should be some ecological explanation for the phenomenon common to all. 

*L. polyedrum* is a red tide organism responsible for massive blooms in surface water that commonly occur off the southern coast of California and Baja. A first consideration is that energy is probably not a limiting factor in growth; photosynthesis probably provides more energy than can be utilized in the relatively slow growing organism. So if excess energy expenditure provides a selective advantage, there is no need to conserve an abundant resource.

However, nitrogen and phosphate are often in short supply, as has been determined from measurements of the sea-water in such blooms. If a protein is functionally important for only a part of the day and another protein is used at a different time, might the amino acids from the degradation of the first be conserved and used for the synthesis of the second? And so forth.

Such an explanation emerged in retrospect from experiments originally carried out by Laura McMurry to test the hypothesis of synthesis and destruction [14]. Cells were grown in a medium with heavy isotopes of carbon and nitrogen for several generations; the luciferase activity in extracts was shown to sediment in a sucrose density gradient at a faster rate, corresponding to the heavier density protein molecules. To determine if the 10-fold increase in activity that occurred in the first cycle after transfer to a normal medium was due to new synthesis of luciferase, the sedimentation velocity of the new activity in extracts was determined by ultracentrifugation.

The results were interpreted as not supporting the hypothesis of synthesis and destruction. More than 50% of the luciferase activity retained the heavy isotope label, more than expected if synthesis and destruction occurred, but less than expected if the luciferase molecules were, for example, reversibly inactivated by an inhibitor of some sort. The results were therefore considered inconclusive and are described only in her Ph.D. thesis [14].

As already noted, subsequent experiments based on antibody measurements established that synthesis and destruction does indeed occur [9,10]. The curious result obtained by heavy isotope labeling might very well be explained by the cellular conservation of amino acids; that is, they are not degraded or lost to the medium, but conserved by the cells for synthesis of new protein in the next cycle. Thus after transfer from a medium with heavy isotopes into one with carbon-12 and nitrogen 14, the amino acids with heavy isotopes derived from the protein degradation would still be available for new protein synthesis.

A later and very tantalizing finding is that two species of a different bioluminescent dinoflagellate genus, *Pyrocystis*, were found to exhibit circadian control of luminescence but not a synthesis and destruction of luciferase; its amount is the same in extracts made during the day and night phases [15]. How the circadian regulation of the bioluminescence in *P. lunula* is accomplished is not known, but it is known that the localization of the luminous organelles (scintillons) from which light is emitted differs from day to night phase [16,17,18].

As an explanation, though not a mechanism, we can speculate that the life styles and ecologies of these two genera differ in a way that gives rise to their differences in the circadian regulation of enzyme activity. For example, bioluminescent members of the genus *Pyrocystis* have been reported to have maximal population densities at depths of 60–100 m, where nitrogen is more abundant than in surface waters, and to undergo diel vertical migration [19]. 

Vertical migrations in a stratified ocean may allow the dinoflagellates to balance cellular energy and nutrient requirements by taking up nutrients (primarily nitrate and ammonium) in deeper waters and migrating to regions with higher photon flux during the day [20]. The vertical migration and nitrogen acquisition by *Pyrocystis* may be a strategy to reduce nitrogen limitation in nature and as a result reduce the selective pressure to recycle nitrogen within the cells through the daily synthesis and degradation of luciferase. The relative contribution of these different strategies of bioluminescence to increased fitness merits further investigation. 

## 5. Conclusions

The fact that many circadian-regulated proteins in the dinoflagellate *Lingulodinium polyedrum* are synthesized and destroyed daily may represent a mechanism to conserve nitrogen. Amino acids released from the hydrolysis of one protein could be available for synthesis of different proteins over the course of the circadian cycle. That cells of a different species, *Pyrocystis lunula*, which also exhibit a circadian rhythm of bioluminescence, do not destroy and resynthesize luciferase, may be related to their ecology; they exhibit a daily vertical migration to deeper water where nitrogen may be readily available.
